# Supplementary material for: Molecular characterization of plant growth-promoting vermi-bacteria associated with Eisenia fetida gastrointestinal tract
Source: PLoS One. 2022 Jun 15;17(6):e0269946. doi: 10.1371/journal.pone.0269946 (PMC9200293; doi:10.1371/journal.pone.0269946)
Supplement: S1 Data — (DOCX) [file pone.0269946.s001.docx]

**Supporting Data**

URLs for nucleotide sequences on NCBI:

<https://www.ncbi.nlm.nih.gov/nuccore/OL364179>

<https://www.ncbi.nlm.nih.gov/nuccore/OL364177>

<https://www.ncbi.nlm.nih.gov/nuccore/OL364180>

<https://www.ncbi.nlm.nih.gov/nuccore/OL364184>

<https://www.ncbi.nlm.nih.gov/nuccore/OL364181>

<https://www.ncbi.nlm.nih.gov/nuccore/OL364187>

<https://www.ncbi.nlm.nih.gov/nuccore/OL364182>

<https://www.ncbi.nlm.nih.gov/nuccore/OL364178>

<https://www.ncbi.nlm.nih.gov/nuccore/OL364185>

<https://www.ncbi.nlm.nih.gov/nuccore/OL364186>

<https://www.ncbi.nlm.nih.gov/nuccore/OL364183>
