# Supplementary material for: Molecular characterization of plant growth-promoting vermi-bacteria associated with Eisenia fetida gastrointestinal tract
Source: PLoS One. 2022 Jun 15;17(6):e0269946. doi: 10.1371/journal.pone.0269946 (PMC9200293; doi:10.1371/journal.pone.0269946)
Supplement: Attachment — Submitted filename: PlosOne Review Comments.docx [file pone.0269946.s002.docx]

Reviewer Comments;

Title: Appropriate

Abstract:

Rephrase the sentence, “Results revealed that 11 vermi-bacterial isolates were isolated from the digestive tract of *Eisenia fetida*.”

Change “Indole acetic acid” to “indole acetic acid”

Rephrase the statement “It was concluded that all vermi-bacterial isolates could be used as potential microbial biofertilizers for the cultivation of crops in Pakistan.”

Abstract is poorly written, needs improvements

Introduction:

Provide latest citations/references

Material and Methods:

Ethical statement: ethical approval from institutional ethics committee is missing

Results:

Table 1: Scientific names must be on standard format “*E. fetida*”.

Discussion: Improve discussion, add latest citations

Conclusion: Conclusion is generalized. Provide specific outcomes of this study.

References: cross check references in the text and list provided

Language: improve manuscript for English language and grammatical errors

Comments for Editor:

Accept with major revision
