# Supplementary material for: Molecular characterization of plant growth-promoting vermi-bacteria associated with Eisenia fetida gastrointestinal tract
Source: PLoS One. 2022 Jun 15;17(6):e0269946. doi: 10.1371/journal.pone.0269946 (PMC9200293; doi:10.1371/journal.pone.0269946)
Supplement: Attachment — Submitted filename: PlosOne Review Comments.doc [file pone.0269946.s003.doc]

Reviewer Comments;

**Reviewer #1**:

1- The literature review is adequate. **Thanks for positive consideration.**

2- The experimental design is appropriate. **Thanks for positive concern.**

3- The manuscript with sound contribution. **Thanks for positive comment**

4- The writing: It does contain many of the usual grammatical mistakes. **Has been modified**

5- The discussion: Rewrite the last two sentences of the last paragraph. The conclusion was overstated beyond what the data actually support. **Has been modified**

**Reviewer #2**

Title: Appropriate

**Thanks for positive consideration.**

Abstract:

Rephrase the sentence, “Results revealed that 11 vermi-bacterial isolates were isolated from the digestive tract of *Eisenia fetida*.”

**Has been rephrased**

Change “Indole acetic acid” to “indole acetic acid”

**Has been changed**

Rephrase the statement “It was concluded that all vermi-bacterial isolates could be used as potential microbial biofertilizers for the cultivation of crops in Pakistan.”

**Has been rephrased**

Abstract is poorly written, needs improvements

**Has been improved**

Introduction:

Provide latest citations/references

**Has been added**.

Material and Methods:

Ethical statement: ethical approval from institutional ethics committee is missing

**Has been added**

Results:

Table 1: Scientific names must be on standard format “*E. fetida*”.

**Has been modified**

Discussion: Improve discussion, add latest citations

**Has been improved**

Conclusion: Conclusion is generalized. Provide specific outcomes of this study.

**Has been modified**

References: cross check references in the text and list provided

**Has been modified**

Language: improve manuscript for English language and grammatical errors

**Has been improved**

Comments for Editor:

Accept with major revision
